# Editorial: Functional study of novel VUS (variant of uncertain significance) mutations in single-gene inherited disease

**DOI:** 10.3389/fgene.2025.1578736

**Published:** 2025-03-19

**Authors:** Guohua Yang, Hua Zhong, Huiliang Wen, Yiping Shen

**Affiliations:** ^1^ Department of Medical Genetics, School of Basic Medical Science, Wuhan University, Wuhan, China; ^2^ Cancer Epidemiology Division, Population Sciences in the Pacific Program, University of Hawai’i Cancer Center, University of Hawai’i at Mānoa, Honolulu, HI, United States; ^3^ School of Food Science, Nanchang University, Nanchang, China; ^4^ Division of Genetics and Genomics, Boston Children’s Hospital, Harvard Medical School, Boston, MA, United States; ^5^ Hejun College, Synergene (Jiangxi) Education, Huichang, Jiangxi, China

**Keywords:** NGS, VUS, functional validation, missense mutation, genetic disease

The field of genetics has witnessed remarkable advancements in recent years. The advent of next-generation sequencing (NGS) technologies has precipitated remarkable progress in our understanding of genetic diseases. Sequencing (WES) and Whole Genome Sequencing (WGS) have revealed numerous variants of uncertain significance (VUS), the pathogenicity of which remains to be determined. Functional validation of these variants is essential for translating genetic findings into clinical practice. The studies featured in this Research Topic collectively examine the clinical relevance of novel VUS by focusing on their functional studies.

A central theme of this Research Topic is the identification and characterization of novel genetic mutations implicated in hereditary diseases. For instance, Wang et al. identified a splicing variant (c.1217 + 2T>A) in the *DEPDC5* gene in familial focal epilepsy with variable foci (FFEVF). Using mini-gene splicing assays the authors demonstrated that this variant disrupts alternative splicing, thereby underscoring the critical role of splicing mutations in epilepsy pathogenesis.

Similarly, Zhang et al. investigated compound heterozygous mutations in *PKHD1,* a gene associated with autosomal recessive polycystic kidney disease (ARPKD). Their study identified two novel mutations, c.3592_3628 + 45del and c.11207T>C, and employed mini-gene splicing assays to confirm the pathogenic effect of the c.3592_3628 + 45del variant. This research provides valuable insights into the genetic architecture of ARPKD and highlights the utility of functional assays in validating novel mutations.

The functional characterization of metabolic disorders also constitutes an important focus of this volume. Wang et al. reported a novel splicing mutation in the *HMBS* gene (c.648_651+1delCCAGG) and verified its pathogenicity through *in vitro* experiments. This mutation leads to abnormal splicing of the *HMBS* gene, resulting in acute intermittent porphyria (AIP). Through protein structure analysis and enzyme activity assays, the study revealed how the mutation induces disease by reducing HMBS expression and activity. This discovery not only enriches our understanding of the pathological mechanisms underlying AIP but also provides a theoretical foundation for future genetic diagnosis and counseling.

The study by Xiao et al. examined VUS in the *MMUT* and *MMACHC* genes in methylmalonic acidemia (MMA) using mass spectrometry and bioinformatics tools. The study found that patients with pathogenic or potentially pathogenic mutations exhibited significantly higher levels of metabolic markers (e.g., C3, C3/C0, and C3/C2) compared to non-mutation carriers. This finding offers a novel approach to assessing the pathogenicity of VUS through metabolic markers and provides a feasible method for screening inherited metabolic disorders.

Qian et al. reported the first Chinese family with TNF receptor-associated periodic syndrome (TRAPS) carrying a rare missense mutation (p.C125Y) in the *TNFRSF1A* gene. The study revealed that this mutation did not cause abnormal receptor shedding but instead triggered an inflammatory response by activating the unfolded protein response (UPR). This discovery provides new insights into the pathological mechanisms of TRAPS and underscores the importance of early genetic diagnosis and personalized treatment.

Wang et al. reported two adjacent missense mutations in the *DYSF* gene (c.5628C>A and c.5633A>T) in a Chinese family associated with muscular dystrophy. By integrating *in vivo* and *in vitro* experiments, they demonstrated that these mutations adversely affect mRNA splicing, elucidating the molecular underpinnings of muscular dystrophy. This study is the first to confirm that double mutations in the *DYSF* gene are associated with the onset of muscular dystrophy and reveals the critical role of the c.5628C>A mutation in splicing function. This discovery not only enriches the mutational spectrum of the *DYSF* gene but also provides a new reference for the clinical diagnosis of muscular dystrophy.

Another significant contribution to this Research Topic is the study by Lin et al., which reports a novel mutation in the *MAF* gene associated with congenital cataracts. The authors used WES to identify the c.901T>C (p.Y301H) mutation and validated its presence in affected family members through Sanger sequencing. Their findings expand the mutation spectrum of the *MAF* gene and emphasize the role of transcription factors in lens development and cataract formation.

The studies presented in this volume underscore the critical importance of integrating genomic analyses with functional assays to unravel the molecular basis of hereditary diseases. They illustrate the power of genomic testing—particularly through Whole Exome Sequencing (WES), Sanger sequencing, and functional assays—to identify and validate disease-causing mutations. Such approaches hold profound implications for genetic counseling and early diagnosis, enabling clinicians to provide more accurate prognoses and personalized treatment strategies.

A key step in the functional study of gene mutations involves constructing appropriate mutation models tailored to specific mutation sites and genes. These models, ranging from cellular systems to organismal models, are essential for verifying the functional impact of genetic mutations and pinpointing variant pathogenicity. As genomic medicine advances, we anticipate the development of more sophisticated and applicable gene mutation models that will not only expand our comprehension of genetic diseases but also offer valuable insights for clinical practice.

Functional validation of missense mutations remains a significant challenge. Although approximately 20% of missense mutations are pathogenic, elucidating the precise mechanism can be difficult, in part because it may be hard to conceive that a single amino acid substitute could be deleterious. Furthermore, some genes have long coding DNA sequences (CDS), making the functional verification of missense mutations requires even more complex. Establishing appropriate models is necessary, but also too are advanced technologies such as cell immortalization, induced pluripotent stem cells (iPSC), gene editing, transposon system, and patch clamp technology. It is hoped that continued methodological innovations will enhance our capacity to functionally characterize missense mutations in the future.

In conclusion, the articles in this Research Topic underscore the transformative impact of genetic research on our understanding of rare genetic diseases. By identifying novel mutations and elucidating their functional consequences, these studies contribute to the growing body of knowledge in genetics, paving the way for more precise and effective medical interventions. We look forward to further research that will advance this area and ultimately improve patient outcomes.

